# “It Is Utterly Out of My Expectation”-A Case Inquiry of Teacher Identity of an EFL Teacher in a Chinese Shadow School Setting

**DOI:** 10.3389/fpsyg.2021.760161

**Published:** 2021-10-15

**Authors:** Feng Wang, Jingwen Guo, Bin Wu, Zhong Lin

**Affiliations:** ^1^School of Foreign Languages, Xidian University, Xi'an, China; ^2^Kang Chiao International School Xi'an Qujiang Campus, Xi'an, China; ^3^School of Foreign Languages, Chang'an University, Xi'an, China

**Keywords:** teacher identity, identity formation, EFL teacher, professional development, shadow school

## Abstract

Though teacher identity is currently receiving an increasing amount of attention in the literature on teacher education and teacher development, little information is available about the richness, fluidity and individuality of EFL teacher identities in various L2 settings. This preliminary case inquiry has echoed that comment by exploring qualitatively how language teacher identity features in the participant's categorization of herself as a professional during her school-to-work transition at a shadow school in hinterland China. Drawing on semi-structured interviews and reflection essays, the triangulated data were analyzed with thematic analysis in order to find categories of enacted identities and key factors that impacted Jane's teacher identity formation. The results indicate four identities existed: an attendant, a firefighter, a coolie and a tramp. The fundamental predicaments hovering over the green employer encompass an array of contextual factors, including (1) overwhelming anxieties for potential early student leavers; (2) overhuge workload and fluid working schedule; (3) endless non-teaching related commitments; (4) lack of career prospect and development ladder. The results of the study contribute to the understanding of the complex and dynamic nature of teacher identity influenced by sociocultural landscapes where language teachers are situated. The research suggests implications for teacher educators and stakeholders on how to transform a novice to a qualified EFL teacher within the ideology and discouraging discourse of a burgeoning privately-owned training market, and on how to mediate green teachers' agency and autonomy against the bottlenecks of their initial years of teaching.

## Introduction

New entrants in their initial teaching years encounter a plethora of difficulties (Ruohotie-Lyhty, 2013). Novice teachers in traditional schools commonly stay in “an insecure job situation” (Ruohotie-Lyhty, 2013), needless to say those in privately-owned shadow schools. However, to become and remain “secure” requires more than learning or adapting specific instructional strategies (Cross Francis et al., 2018). To gain a stable teacherhood necessitates transformative changes across affective, cognitive and motivational dimensions. Given this perspective, it is paramount to guide and help new employees in shadow schools to navigate “induction” during the early years of teaching and negotiate the discrepancy between their personal and professional selves.

Technically, teacher professional selves are termed as teacher identity, which is broadly defined as the way teachers “make sense of selves and the image of selves that they present to others” (Day, 2011, 48). Over the past decades teacher identity has gained growing interest in teacher education (Beauchamp and Thomas, 2009; Yuan and Mak, 2018). The construct was assumed to be a critical research “lens” (Gee, 2000) to provide insights into the complex process of growing from a novice to an experienced teacher because choosing teaching as a career encompasses dimensions of individual qualities, expectations, values, beliefs, and talents (Richardson and Watt, 2006), as well as accommodating required social roles, responsibilities, policies, and classroom environments.

## Literature Review

“Identity” has been conceptualized from different disciplinary perspectives by researchers from philosophy, psychology, anthropology, sociology, and neuro-science, among others. A vast and varied literature indicates that identity is not a singular construct (Richardson and Watt, 2006), and specific subcategorized identities, such as gender, ethnicity, spirituality, profession, are enacted and experienced by individuals in sociocultural contexts through lifespan. Identity is also dynamic in that a mutation in physiological, psychological, and biological needs, and together with sociopolitical forces, may result in identity shift and growth.

Educators develop this construct by emphasizing the need to balance “self and other” to frame as individuals (see Erickson, 1968). Kegan's (1982) viewpoint of identity appeared to be most relevant to this study, as he held identity construction as a constant balancing and re-balancing of “self and other” in a way that resembles a spiral or double helix; in this context, one person revisits similar challenges at different points of his or her life, and “with divergent circumstances comes to understand these challenges, and themselves, more deeply” (Alsup, 2006), which psychologist Kroger (2004) confirmed as “breaking through” by spending much of a person's life being developmentally “out of balance,” and moving from one state of subject-object balance to another.

Subsumed into profession identity, teacher identity demonstrates how teachers sense, understand and make meaning regarding what they do during the process of professional development (Beijaard et al., 2004), and it is a label that describes how teachers seem to understand themselves as teachers. Teacher identity may greatly impact teacher education and language education in that it plays an important role in an individual's teaching not only in terms of how he/she teaches but also how he/she presents themselves and the materials for teaching and learning. Consequently, the teacher identity has attracted extensive attention in social sciences since the 1950s. The study of the identity of language teachers, ever since the mid-1990s, has gradually entered the field of language teaching or TESOL. Antonek et al. (1997) and Duff and Uchida (1997) studies can be regarded as pioneering research in this regard. Ever since, more researchers paid attention. Over the past 10 years, positive experiences of language teachers' identity construction in their specific contexts have been increasingly reported. Their findings revealed work experiences of language teachers in influencing their teaching and self-perceptions.

As suggested by theorists, teachers' identity development is a social balancing and rebalancing, which is tied to teachers' cognitive and emotional engagement with the contextualized community (Antonek et al., 1997; Duff and Uchida, 1997). According to Sachs (2005), teacher identity is essential to the teaching profession as “it provides a framework for teachers to construct their ideas of “how to be,” “how to act,” and “how to understand” their work and their place in society” (p. 15). It often refers to “the way we make sense of ourselves and the image of ourselves that we present to others” (Day, 2011, p. 48). Gee (2000) claims that “identity suggests the “kind of person” one is within a particular context” (p. 99). Olsen (2011) further argued that identity is a reflection of the influences teachers have gained from the settings and the various intertwined components of constructs such as self and social positioning.

Underlying the teacher identity is the sociocultural and sociopolitical factors (Varghese et al., 2005). Current studies about teacher identity issues pointed out that teachers are not separated from the environment, and the evolution of teacher identity is tightly related to the “affordances” of their external environment (Ruohotie-Lyhty, 2011; Pappa et al., 2017). Specifically, teachers' professional identity has been seen as an essential factor in representing teachers' professional lives which largely affect “teaching quality, job motivation; commitment and resilience and career decision-making” (e.g., Van den Berg, 2002; Day and Hong, 2016).

A synthesis of previous research on teacher identity construction indicates three territories of research interest. The first one is the multifaceted nature of identity transition and the interwoven interrelationship between these dimensions (e.g., Trent, 2016; Chang, 2018). The second concerns the correlation between the personal and social dimensions of identity construction. Most enquiries of this line target the personal aspects, emphasizing self-reflection on who one is, what one aspires to become as well as teachers' practical knowledge (e.g., Antonek et al., 1997). The last, closely related to the second, goes to the relationship between autonomy and structure in identity construction (e.g., Coldron and Smith, 1999).

Discernibly, the bulk of existing literature has examined identity development, identity conflicts and struggle, and identity crisis experienced by L2 language teachers. Given that the main language teaching force in L2 context work at traditional public schools, the existing literature has ignored the contextual complexity faced by EFL teachers in shadow schools. As reviewed by Moussu and Llurda (2008), little research was conducted to tap the richness, fluidity and individuality of EFL teacher identities in various L2 settings beyond the traditional, externally prescribed framework (native and non-native). To the gap, this present study attempts to illuminate the features of an intriguing process of teacher identity construction through one shadow-school teachers' 3-year teaching experiences in light of her social, cultural, and emotional engagement. The study aims to demonstrate the critical, intricate and unique enacted roles by providing a more thorough understanding of the ways in which a green teacher encountered her challenging situations and in response, built her identities. Also, this study contributes to the knowledge and understanding of the “murky” and “unplugged” (Barkhuizen, 2016) concept of language teacher identity. The results of this study may therefore suggest practical implications for those involved in designing and implementing teacher education programmes and stakeholders to help teachers of varied backgrounds experience a positive process of identity formation and boost their professional competence and commitment. The research questions addressed in the paper involve (1) What identities did Jane enact during her teaching experience at the shadow school? (2) Why and how did these identities project?

## Methodology

Through convenience sampling and purposive sampling, Jane (pseudonym) was invited to participate in this study. We firstly approached seven English teachers in several shadow schools and found that only Jane, just started her teaching experience (a typical novice teacher), and she was also interested in the topic of teacher identity (cooperativeness). Jane was born in 1994 and she was raised in Xinjiang Uygur Autonomous Region which is around 2,500 kilometers away from Xi'an City. In 2013, she became an undergraduate student in the Foreign Studies School in X University, a top 100 project University in China, majoring in English Language and Literature. She graduated from the University in 2017. As a channel for providing job positions, shadow schools have mushroomed over the last two decades against the background of the fierce competition of exam-oriented culture in China. Consequently, University graduates choose to work in these institutions for higher pays compared with that in the traditional school. Jane did not escape from the tide. Upon graduation Jane was employed as an English teacher at the shadow school located in Xi'an, a hinterland city in Northwestern China. At the time of data collection, Jane has just 1-month teaching experience in this shadow school.

The site of the study is a shadow school located at a downtown business center. It was set up in 2000, and when the research program began, it owned 46 staff with 80 plus registered students ranging from primary pupils to senior secondary school students most of whom come to have English lessons out of the school time. All the three interviews were conducted in the Jane's office at the shadow school.

This study employs a case study approach (Buston et al., 1998). A case study, as one of the common methods of qualitative research, is a bounded system and a phenomenon of some sort of occurring in a bounded context (Miles and Huberman, 1984, p. 25). Case study inquiry is appropriate here because the present study context fits the definition of a bounded case in the sense that it emblematizes the convergence of a particular time and space-the reflections by Jane on her teacher development at a shadow school spanning 3-plus years. Data for this preliminary case study were collected over the course of the research program.

A semi-structured interview was employed to collect data. Thirty questions was designed in Chinese (the first language of Jane), so as to draw forth “inner voice” of the participant (DeVault, 1990). With consent of the participant, the study recorded interviews. The thirty questions designed covered job-seeking experiences, teaching experiences, working conditions, and self-reflections, and these questions were asked in chronological sequence. We conducted three sessions of interviews ranging from 25 to 45 min (All data were collected in Chinese), one at the onset of Jane's working, one after the second year and the other at the end of the third year at the shadow school. All of the interviews were recorded with Jane's permission. In the interviews, Jane shared her experiences and feelings about working as an EFL teacher and her working conditions. After that, we transcribed the recordings before triangulated with reflection responses.

Transcribing the recorded interviews is a “common practice” in qualitative research. After the interviews were finished, we transcribed the tape recordings into texture information for a better understanding of the identities of Jane lived by her in her work. When we transcribed the tape recording, we also paid attention to the silences including “many and profound, meanings” (Poland and Pederson, 1998). Pseudo names were used in the study to protect the participants' privacy.

For triangulation of data, multiple measures of data were collected that contained six reflection essays (one for each semester). In the reflection essays, Jane was asked to reflect on her challenges and worries in her teaching, and her development of her teacherhood.

While the reflection essays were processed by one of the authors, the semi-structured interviews were administered and facilitated by the other three. All authors observed lessons given by the research participant, as support and supplementary for teacher reflection and interview data. The data were analyzed with thematic analysis (Braun and Clarke, 2006) in order to identify recurring categories themes of identities and factors that impacted Jane's teacher identity development. A three-round data analysis was made. In the first round, we read the transcription word by word and entered each code when the fragments of transcription showed, depicted or implied a piece of meaning related to the teacher identity. We preferred using Jane's local terminology to name the codes, and we added some memos in the necessary places.

In the second round, we carefully examined the opening codes created in the first round and rigorously compared them through their internal relationships. After that, we observed the basis of career construction theory which identifies three social challenges that prompt change-“vocational development tasks, occupational transitions, and work traumas” (Savickas, 2005) and continued our coding in the second round. The third round of data analysis gave birth to the selective coding which is the tentative classification of the final conceptual framework. Afterwards, the authors compared their analysis and annotations multifarious times so as to gain an agreement of the common categories and factors.

## Findings

As discussed above, the core of an identity is the categorization of the self as an occupant of a role, and the incorporation, into the self, of the meanings and expectations associated with that role and its performance (Burke and Tully, 1977; Thoits, 1986). In the present study, common themes were found throughout the data that describe the participant's attempting to fit in to her shadow school. This section highlights the main findings of the study that are grouped into the following categories: an attendant, a firefighter, a coolie and a tramp. Key factors to impact the participant's teacher identity formation encompass: overwhelming anxieties for potential early student leavers, overhuge workload and fluid working schedule, endless non-teaching related commitments, and lack of career prospects.

### An Attendant: Overwhelming Anxieties for Potential Early Student Leavers

Jane began her teaching thinking of herself as an English instructor but came out of 3 years of classroom practice viewing herself as an attendant, something like a baby-sitter. It makes her suffer overwhelming anxieties for potential early student leavers. “It is utterly out of my expectation” remarked Jane.

In Jane's opinion, teachers who work in shadow schools (profit-oriented and exam-oriented training institutions) are different with those who work in the public schools because they have to pay more attention to the relationship with students rather than the teaching itself. In this way, it is difficult for teachers like Jane to construct their teacher identity because they resemble attendants to students rather than educators.

“*I think teachers need to focus on student achievement, but for a shadow school teacher we have to consider the cognitive response from students. Our students can come and go easily, so we have to give students a feeling of relaxation or closeness.”*

In contrast to other novice teachers who have opportunities to explore the teacher identity and “to test self-perceptions of their developing identity as a teacher” (Pittard, 2003), Jane and her colleagues in this shadow school have to blur the teacher-student boundary to gain more student registration. She argued that teachers in public schools would alienate their students to defend their teacher identity. “They don't have to worry about no classes to give, so they definitely have fewer anxieties than us.” In order to maintain a good relationship with her “clients,” Jane had to be quite cooperative to ensure that students are willing to purchase her courses in the next session.

“*I could not criticize my students for their bad performance in the class, or even for their skipping of handing in homework. I had to try to pretend to be happy even when the class discipline was not in order. I had to try to answer any questions even unrelated to English. Students sometimes discussed the result of a football match of European Football Association or some tidbits of some stars. At that time I have to sacrifice my class time.”*

Although Jane's students never threw her off for a “better” teacher which gives her a sense of accomplishment and satisfaction, she spoke her mind that this kind of relationship brings her a weak teacher identity. And this is just the tip of the iceberg of her uneasy identity transition.

### A Firefighter: Odd Hours and Fluid Working Schedule

“*I'm swamped and tired now. Compared with the teachers in public schools, we have no summer or winter vacations, and we have to work at weekends continually. I had lots of forms to fill in and administration meeting to attend.”*

As a teacher in the shadow school, Jane has to change her schedule to adapt to the fluid mandates of the shadow school. She had 10 classes to teach 1 week, but might 20 another week because students might come to register at any time. The workload is also very provisional for teachers in the shadow school because teachers may quit or students may “drop out” at any time. When one teacher turns over, other teachers have to shoulder his/her workload until new employers come.

“*In the week, working from 9 A.M. to 5 P.M. is my routine. While in the weekend, the work time could have destroyed me, it is from 8 A.M. to 10 P.M. At first I couldn't fit this schedule because even though I leave off at 10 P.M., there still be like … a hundred messages from students or their parents waiting for me.”*

Jane specified that she worked as a coolie who has is all the time responsible for any happenings. She also said the administrators at school see it as “an effective approach” to ensure every student is happy to come and remain in registration. The story of Jane contains a lot of pressure, and her experience shed light on the problems many Chinese novice teachers faced, especially in privately owned schools.

### A Coolie: Endless Non-teaching Related Commitments

If taking teaching responsibilities is the compulsory lesson in Jane's identity transition, being a modern coolie looks like a “nowhere-left-to-run” destiny of her working life.

Transiting identity from a student to a teacher is an uphill struggle for Jane because she had no academic background of education, and this is the sticking point for her to solve when she was a novice teacher because being a teacher is out of her plan.

“*I had never thought about being an EFL teacher because I only concentrated on learning English literature in college. I had no passion for the teaching profession. But when I looked for a job to feed myself, I considered myself to be a teacher. And then, everything pushed me to do it. Like my parents, they are just like many Chinese parents who think teachers' work is stable and easy. But actually, they must be wrong because my work isn't easy as they imagined.”*

Things changed when Jane received compulsory training like “some basic teaching methods” and “how to make a successful writing course,” Jane has gradually accept her identity as a teacher at the shadow school until endless non-teaching related commitments come. The extra-curricular activities make the teachers in the professional positions frazzled and tired both physically and mentally.

“*Although teaching-related works are full of challenges, they have accomplishments. Sometimes I enjoy staying with students because they've helped me find out a better self, like being a patient teacher. But the thing is, currently there are many unnecessary things, as far as I considered, attending more than 7 meetings in a week is one of them. Besides, there's a rule that teachers in our section have to take English language tests every year like IELTS, TOFEL or PET. Who fails the test, who has potential risks to be fired. (She forced a smile.)”*

When the interview question came to “whether sticking to be a teacher at shadow school,” there was a silent time for seconds. Jane holds an ambivalent attitude to the teaching profession.

### A Tramp: Lack of Career Prospects and Development Ladder

There are some factors pointed to influence job seekers' choices like “physical workload; time pressure; recipient contact demands; physical environment; shift work; work-home interference; emotional demands; workload; and cognitive demands” (Scanlan and Still, 2019, p. 3). In Jane's opinion, she considers career prospects and development ladder are significant impact factors for her, though she is trying to figure out them in the present position.

“*Whenever I look back, I feel things happened in our fourth-year (at the university) just like a big sale in the market. The graduates, including me and others, are the products on the shelves and waiting for someone (employers) to pick. The shadow schools were comparable nice “buyers” at that time, so many of us were willing to be put in their bags. But the situation after that til now, you know, is making me consider jumping out.”*

Jane pointed she wants to quit because she can hardly see the future and many her ex-colleagues switched the job because they have no job security and a promise of steady employment.

“You can hardly find teachers who are over 40 years old in our institute,because few of them can bear the times of stress. It is not necessary to do an unfavorable job. Although I don't think about what to do in the future, I just take a step forward. Presently I am a tramp.”

To date, Jane still works for the shadow school. She explained, though in this working condition she might only have limited possibilities to fulfill herself, she wants to fit the identities here and then awaiting a better chance.

## Discussion

In contrast to most previous studies of teacher identity, which investigated teacher identity at traditional schools with semi-structured interviews, this shadow school based study adopted interviews combined with reflection responses and classroom observation to examine how teacher identity features in a privately owned institution and the underlying factors impacting teacher identity formation. It was found that Jane experienced her teacher identity formation by negotiating between different roles, expectations, interests and demands as a professional during her school-to-work transition. The findings show that Jane's engagement with her community plays a great part in informing her identity. Such findings provide insight into the richness and complex nature of teacher identity in the context of a burgeoning training market and illustrate how teacher educators and stakeholders should help new employer in shadow schools navigate through. In this section, we turn back to the questions of the present study: What identities did Jane enact during her teaching experience at the shadow school? Why and how did these identities project?

While a number of studies have examined positive experiences of language teacher identity and provided many insightful implications (Said, 2014; Song, 2016; Chan and Lo, 2017), it is difficult to transform these findings into every actual teaching practice, especially into shadow schools, which are highly contextualized and individualized. This shadow school-oriented preliminary case study found that in an actual teaching context, the instructor tended to undergo various identity conflicts and struggles, hence enacting negative identities. This finding suggests that we should examine the nature and factors in more specific and varied teaching contexts where they are embedded, although some studies have been significant in understanding language teacher identity at intrapersonal and interpersonal levels (Trent, 2015).

Drawing upon the analytical framework of thematic analysis (Braun and Clarke, 2006), this study indicates that Jane in the shadow school took on four identities which are quite different from her subjective perceptions of teacher identity. For her, to be a teacher meant a stable lifelong job, a regular schedule with nine-to-five model, a regular classroom organizer and a steady climber on the social ladder. However, things are quite different: she has to fit in to the shadow school so as to categorize herself as four “negative” identities. The finding reinforced the previous studies (e.g., Choe, 2016; Chang, 2018; among others) in that there are various influencing factors, including school policy and mandates, curriculum and educational system, performative culture characterized by accountability and competition, teacher's individual agency and English proficiency. Jane's tale of the present study can be attributed to China's deep-seated exam-oriented culture, an epitome of curriculum mandates and classroom reality in the fierce out-of-school English training market in China.

Another important finding of the research is teacher identity formation is closely related to career adaptability. Savickas (2005) has conceptualized career construction theory as a “holistic framework” to review one's life experiences in a narrative way, and the career adaptability as one part in the CCT can help us find individual's “inner needs and outer opportunities into harmony” (Savickas et al., 2009, p. 1). The career adaptability which is the third center component in the career construction theory and this can help us analyse the identity transition of teachers in shadow schools because it involves one's adaptation to transitions (Savickas, 2005). Teachers like Jane can obtain a successful identity transition by enhancing their career adaptability. Also, we can solve problems like teacher attrition by increasing teachers' career adaptability. The career adaptability has four dimensions: “confidence,” “exploration,” “planning,” and “career decision making” (Savickas, 1997, 2002), so the difficulties of Jane which also haunt numerous EFL teachers in Chinese shadow schools can be solved by applying this theory.

Firstly, EEL teachers like Jane need to build up confidence in the workplace, because self-perception can change one's identity as they change the views of themselves (Del Corso and Rehfuss, 2011). Confidence gives teachers a sustainable working condition and to help them establish an authority figure in the workplaces during the transitional status. Also, being confident can help teachers deal with relationships with students, because they may have communications with their students frequently. For example, they may answer students' questions without hesitation and nervousness. In this way, confidence helps them build a good relationship with students in the long term.

Secondly, teachers should make a reasonable plan for themselves and their administrators should make plans for better management in the shadow school. For teachers, they need to plan what they want to do in a short-term, and they need to know what their long-term goals are. In such a way, they can complete their work efficiently, and it can also help them release their stress in their positions. For administrators, the management plans should focus on giving administrative support to teachers which allow teachers to get motivation in every aspect of work. The administrators' plans should be encouraging and supportive, and teachers can effortlessly work in their positions. For instance, administrators in the shadow school can adjust the workload of teachers, which allows teachers more time to adapt to their career.

Thirdly, teachers who work in shadow schools need to explore their career, because this can help them find job satisfaction and interests in the teaching profession. Teachers can acquire more professional knowledge by teacher education and training because the effectiveness of teacher education and training can help teachers build their identities and catering the career of professional teaching in the earlier stage. For example, systematic training like Jane mentioned in the interview can help teachers to establish their role in the classroom and complete classes perfectly. In this way, exploring in the teaching profession can bring teachers a sense of satisfaction which can help people to evaluate their contributions in the work and to fit into their social niche (Erikson, 1968).

Lastly, teachers should make the right decision-making during their identity transition thought they may doubt themselves and the teaching profession in this period, because it may influence their career development. Before making decisions, they need to consider inner reasons such as families, psychological conditions and so on, and they need to consider the external factors such as working conditions, relationships with colleagues and so on. For example, when Jane wants to give up, she needs to think about the motivation as an EFL teacher. If she has no interests in teachers, there is no need for her to stay in shadow schools.

## Conclusion

Understanding teacher identity-related dilemmas and their diverse significance for teaching and learning can help “equip language education for exciting social changes as well as daunting global challenges” (Nelson, 2016). This study reveals four negative identities enacted by Jane who was employed in a shadow school, and some applications are suggested for teacher identity formation to enhance their career adaptability and complete their identity transition.

There are four issues exposed by Jane's narrative. First, the EFL teachers work in the profit-oriented schools may find difficulties in dealing with the relationships with students. Second, the workload in shadow schools influences teachers' life, and it makes them struggle in transitional status. Third, Jane and other teachers can hardly adapt to their teacher identity. Fourth, they are confused about their career constitution and career planning.

## Data Availability Statement

The raw data supporting the conclusions of this article will be made available by the authors, without undue reservation.

## Ethics Statement

Ethical review and approval was not required for the study on human participants in accordance with the local legislation and institutional requirements. The patients/participants provided their written informed consent to participate in this study.

## Author Contributions

FW and ZL designed and revised the paper. BW revised the first draft of the paper. JG collected data and wrote the manuscript. All authors contributed to the article and approved the submitted version.

## Conflict of Interest

The authors declare that the research was conducted in the absence of any commercial or financial relationships that could be construed as a potential conflict of interest.

## Publisher's Note

All claims expressed in this article are solely those of the authors and do not necessarily represent those of their affiliated organizations, or those of the publisher, the editors and the reviewers. Any product that may be evaluated in this article, or claim that may be made by its manufacturer, is not guaranteed or endorsed by the publisher.
